# Supplementary material for: Oxamniquine derivatives overcome Praziquantel treatment limitations for Schistosomiasis
Source: PLoS Pathog. 2023 Jul 10;19(7):e1011018. doi: 10.1371/journal.ppat.1011018 (PMC10359000; doi:10.1371/journal.ppat.1011018)
Supplement: S1 Fig — A. OXA against S. mansoni. B.1. CIDD-0149830 against S. mansoni, B.2. CIDD-0149830 against S. haematobium. B.3. CIDD-0149830 against S. japonicum. C.1. CIDD-0150610 against S. mansoni, C.2. CIDD-0150610 against S. haematobium and C.3. CIDD-0150610 against S. japonicum. D.1. CIDD-0150303 against S. mansoni, D.2. CIDD-0150303 against S. haematobium, and D.3. CIDD-0150303 against S. japonicum. E. The percentage of worms killed at each concentration. OXA and OXA derivatives were tested against adult male worms. All drugs were solubilized in 100% DMSO. All screens were performed in experimental and biological triplicate. Survival was plotted as a percentage over time using Prism/Curve Comparison/ Long-rank (Mantel-cox) test. The p-value threshold for each derivative compared to DMSO was <0.001. (DOCX) [file ppat.1011018.s001.docx]

**
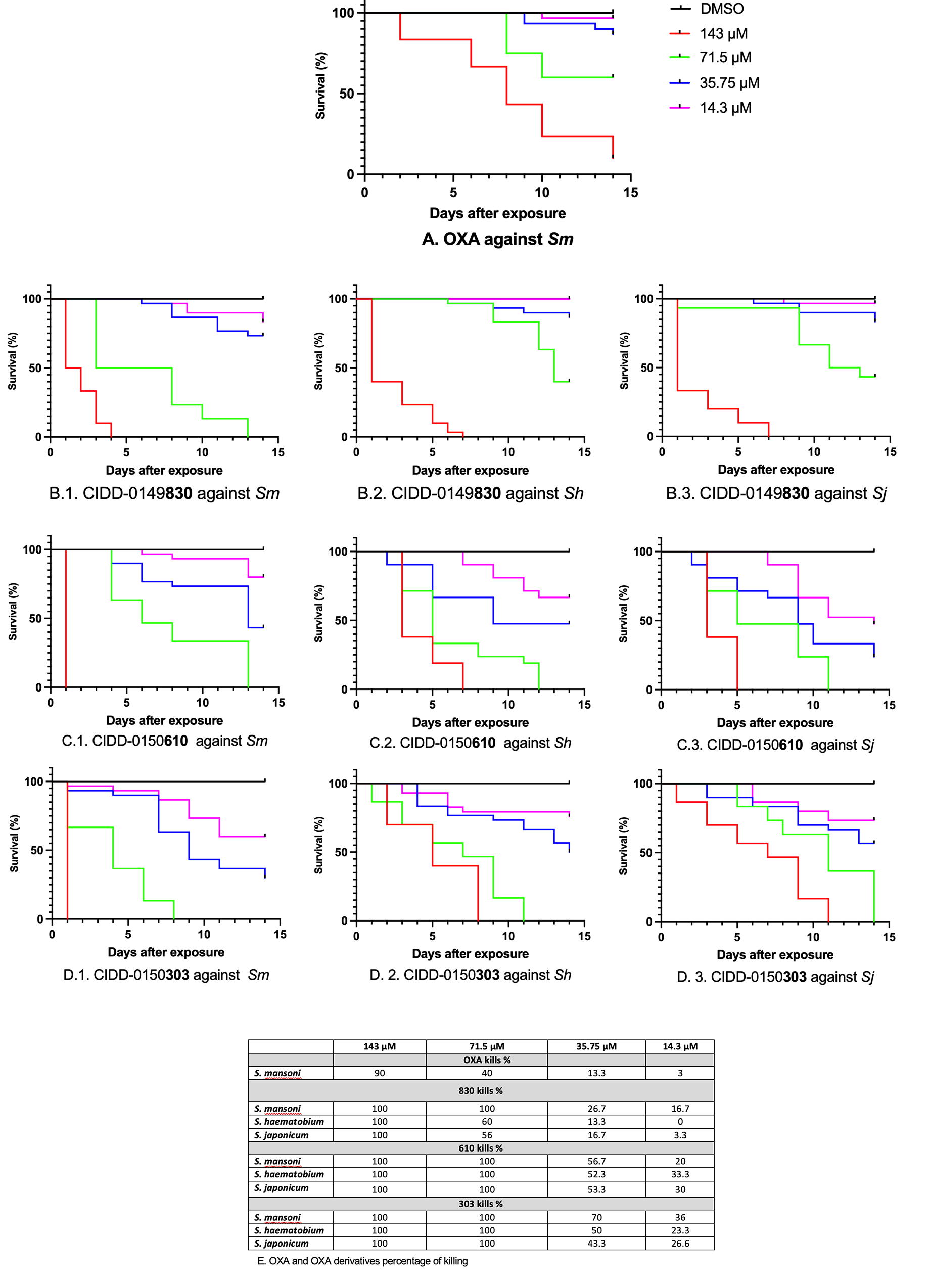
**

**S1_Fig. Kaplan-Meier Curves Demonstrate the Ability of OXA And OXA Derivatives to Kill *Schistosoma* Species at Final Concentrations of 143 µm, 71.5 µm, 35.75 µm, And 14.3 µm Per Well *In Vitro.*** A. OXA against *S. mansoni*. B.1. CIDD-0149830 against *S. mansoni*, B.2. CIDD-0149830 against *S. haematobium.* B.3. CIDD-0149830 against *S. japonicum.* C.1. CIDD-0150610 against *S. mansoni*, C.2. CIDD-0150610 against *S. haematobium* and C.3. CIDD-0150610 against *S. japonicum.* D.1. CIDD-0150303 against *S. mansoni*, D.2. CIDD-0150303 against *S. haematobium, and* D.3. CIDD-0150303 against *S. japonicum.* E*.* The percentage of worms killed at each concentration. OXA and OXA derivatives were tested against adult male worms*.* All drugs were solubilized in 100% DMSO. All screens were performed in experimental and biological triplicate. Survival was plotted as a percentage over time using Prism/Curve Comparison/ Long-rank (Mantel-cox) test. The p-value threshold for each derivative compared to DMSO was <0.001.
