# Supplementary material for: Oxamniquine derivatives overcome Praziquantel treatment limitations for Schistosomiasis
Source: PLoS Pathog. 2023 Jul 10;19(7):e1011018. doi: 10.1371/journal.ppat.1011018 (PMC10359000; doi:10.1371/journal.ppat.1011018)
Supplement: S2 Fig — mansoni Sulfotransferase Confers Resistance Upon Challenge. A.1. CIDD-0149830: SmSULT RNAi alone, Irrelevant RNAi, SmSULT RNAi + OXA, and SmSULT RNAi + 830 had 93%+ survival and were displaying healthy characteristics. All other groups expressed similar, expected sensitivity levels to 830 treatments A.2. CIDD-0150610: SmSULT RNAi alone, Irrelevant RNAi, and SmSULT RNAi + 610 had 90%+ survival and were displaying healthy characteristics. All other groups expressed similar, expected sensitivity levels to 610 treatments A.3. CIDD-0150303: SmSULT RNAi alone, Irrelevant RNAi, and SmSULT RNAi + 303 had 93%+ survival and were displaying healthy characteristics. All other groups expressed similar, expected sensitivity levels to 303 treatments. (DOCX) [file ppat.1011018.s002.docx]

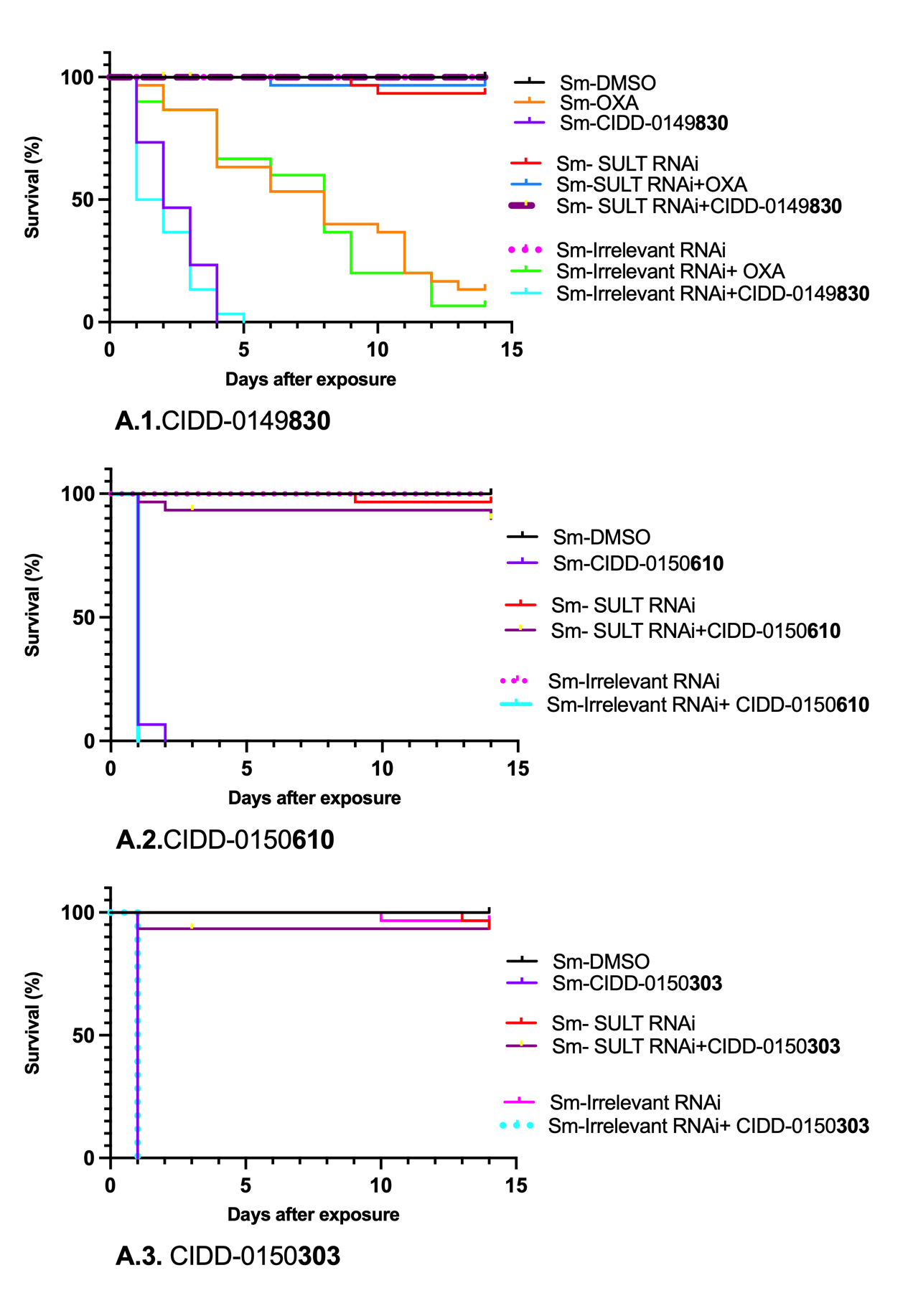


**S2_Fig**. **Kaplan-Meier Curves Demonstrate the Knockdown of *S. Mansoni* Sulfotransferase Confers Resistance Upon Challenge.** A.1. CIDD-0149830**:** *Sm*SULT RNAi alone, Irrelevant RNAi, *Sm*SULT RNAi + OXA, and *Sm*SULT RNAi + 830 had 93%+ survival and were displaying healthy characteristics. All other groups expressed similar, expected sensitivity levels to 830 treatments A.2. CIDD-0150610: *Sm*SULT RNAi alone, Irrelevant RNAi, and *Sm*SULT RNAi + 610 had 90%+ survival and were displaying healthy characteristics. All other groups expressed similar, expected sensitivity levels to 610 treatments A.3. CIDD-0150303**:** *Sm*SULT RNAi alone, Irrelevant RNAi, and *Sm*SULT RNAi + 303 had 93%+ survival and were displaying healthy characteristics. All other groups expressed similar, expected sensitivity levels to 303 treatments.
