# Supplementary material for: Oxamniquine derivatives overcome Praziquantel treatment limitations for Schistosomiasis
Source: PLoS Pathog. 2023 Jul 10;19(7):e1011018. doi: 10.1371/journal.ppat.1011018 (PMC10359000; doi:10.1371/journal.ppat.1011018)
Supplement: S1 Table — (DOCX) [file ppat.1011018.s003.docx]

**S1_Table. Chemical Structure Of CIDD-066790,** **CIDD-0149830, CIDD-0150610, and CIDD-0150303.**

| **OXA derivatives** | **Chemical Structure** |
| --- | --- |
| CIDD-066**790** | 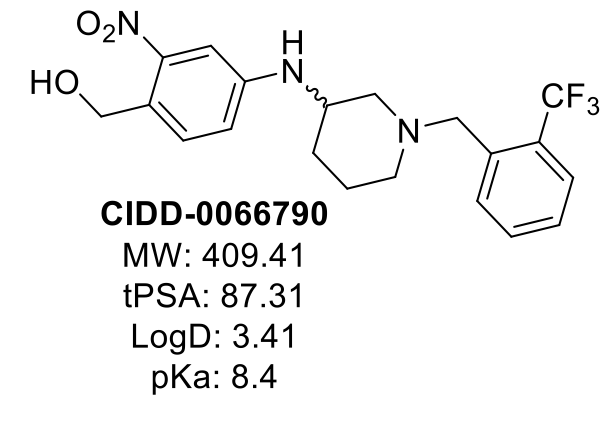 |
| CIDD-0149**830** | 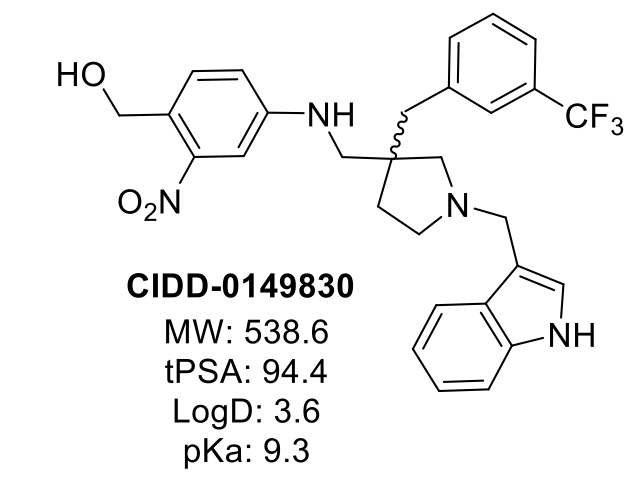 |
| CIDD-0150**610** | 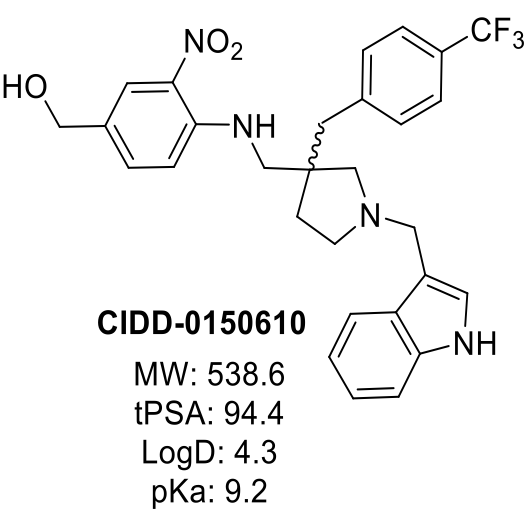 |
| CIDD-0150**303** | 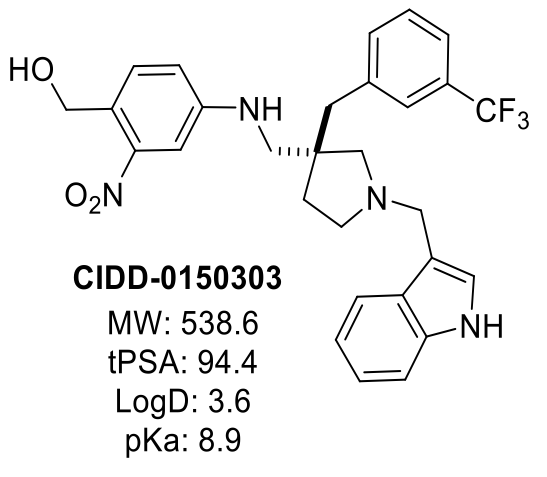 |
