# Supplementary material for: Oxamniquine derivatives overcome Praziquantel treatment limitations for Schistosomiasis
Source: PLoS Pathog. 2023 Jul 10;19(7):e1011018. doi: 10.1371/journal.ppat.1011018 (PMC10359000; doi:10.1371/journal.ppat.1011018)
Supplement: S2 Table — (DOCX) [file ppat.1011018.s004.docx]

**S2_Table.** **Crystallographic Data Collection and Refinement Statistics.**

| **Data collection** |  |  |
| --- | --- | --- |
| PDB Code | 8E5Q | 8E5R |
| Ligands | CIDD-0150303 | CIDD-0150610 |
| Space group | *P*2_1_2_1_2_1_ | *P*2_1_2_1_2 |
| Cell dimensions |  |  |
| *a*, *b*, *c* (Å) | 46.8, 58.5, 90.5 | 140.9, 39.7, 53.8 |
|    γ  | 90, 90, 90 | 90, 90, 90 |
| Wavelength (Å) | 0.97918 | 0.97918 |
| Resolution (Å) | 90.53-1.33 (1.40-1.33)* | 140.89-1.40 (1.48-1.40) |
| *R*_pim_ | 0.025 (0.785) | 0.022 (0.817) |
| CC_1/2_ | (0.557) | (0.515) |
| Mean * * ** | 13.6 (1.0) | 13.3 (1.0) |
| Completeness (%) | 99.6 (98.7) | 97.6 (99.9) |
| Redundancy | 6.2 (6.3) | 6.0 (6.2) |
| Wilson value (Å^2^) | 18.3 | 23.7 |
| **Refinement** |  |  |
| Resolution (Å) | 49.13-1.33 (1.36-1.33) | 53.84-1.40 (1.44-1.40) |
| No. reflections | 57,487 | 58,966 |
| *R*_work_ / *R*_free_ | 0.182/0.217 | 0.169/0.199 |
| No. atoms |  |  |
| Protein | 2,015 | 2,113 |
| Ligands | 66 | 84 |
| Solvent | 215 | 222 |
| *B*-factors (Å^2^) |  |  |
| Protein | 32.5 | 33.4 |
| Ligand | 34.5 | 41.5 |
| Solvent | 40.0 | 41.6 |
| R.m.s. deviations |  |  |
| Bond lengths (Å) | 0.007 | 0.013 |
| Bond angles () | 1.049 | 1.247 |
| Ramachandran Plot |  |  |
| Favored (%) | 96.62 | 96.84 |
| Allowed (%) | 3.38 | 3.16 |
| Outliers (%) | 0.00 | 0.00 |

*Values in parentheses are shown for the highest resolution shell.
