# Supplementary material for: Oxamniquine derivatives overcome Praziquantel treatment limitations for Schistosomiasis
Source: PLoS Pathog. 2023 Jul 10;19(7):e1011018. doi: 10.1371/journal.ppat.1011018 (PMC10359000; doi:10.1371/journal.ppat.1011018)
Supplement: S3 Table — (DOCX) [file ppat.1011018.s005.docx]

**S3_Table. OXA Derivatives Against Schistosoma Species *In Vitro* Results**

|  | **OXA** | **830** | **610** | **303** |
| --- | --- | --- | --- | --- |
| Killing *Schistosoma* species | *S. mansoni* | *S. mansoni*  *S. haematobium*  *S. japonicum* | *S. mansoni*  *S. haematobium*  *S. japonicum* | *S. mansoni*  *S. haematobium*  *S. japonicum* |
|  | | | | |
| Dose require to kill 100% of *S. mansoni* | 143 µM kills 90% | 71.5 µM | 71.5 µM | 71.5 µM |
| Dose require to kill 100% of *S. haematobium* | Not effective | 143 µM | 71.5 µM | 71.5 µM |
| Dose require to kill 100% of *S. haematobium* | Not effective | 143 µM | 71.5 µM | 71.5 µM |
|  | | | | |
| Killing adults of single sex- female and male worms and female and male worms in worm pairs of ***S. mansoni*** in 143 µM | Effective killing for both gender except paired males 60% of killing | 100% of killing of both gender except  paired females 60% of killing | 100% of killing of both gender | 100% of killing of both gender |
| Killing adults of single sex- female and male worms and female and male worms in worm pairs of ***S. haematobium*** in 143 µM | Not effective | 100% of killing of both gender except  paired females 63% of killing | 100% of killing of both gender | 100% of killing of both gender |
| Killing adults of single sex- female and male worms and female and male worms in worm pairs of ***S. japonicum*** in 143 µM | Not effective | 100% of killing of both gender except  paired females 70% of killing | 100% of killing of both gender | 100% of killing of both gender |
|  | | | | |
| Killing juvenile worms of ***S. mansoni*** in 143 µM | Not effective (data not shown) | 100% of killing | 100% of killing | 100% of killing |
|  | | | | |
| Killing **PZQ-resistant strain** in 143 µM | Not tested | 100% of killing | 100% of killing | 100% of killing |
