# Supplementary material for: Oxamniquine derivatives overcome Praziquantel treatment limitations for Schistosomiasis
Source: PLoS Pathog. 2023 Jul 10;19(7):e1011018. doi: 10.1371/journal.ppat.1011018 (PMC10359000; doi:10.1371/journal.ppat.1011018)
Supplement: S4 Table — (DOCX) [file ppat.1011018.s006.docx]

**S4_Table. Test The Efficacy of OXA Derivates in An *in Vivo* Model.**

| A-The reduction in worm burden after treatment with OXA derivatives against *Schistosoma* species (5 animals were treated with a single dose by oral gavage with 100 mg/kg) | | | | | |
| --- | --- | --- | --- | --- | --- |
|  | **OXA** | **830** | **610** | **303** | **790** |
| *S. mansoni* | 93% | 72.3% | 47% | 81.8% |  |
| *S. haematobium* | Not effective | 80.2% | 69.1% | 60% |  |
| *S. japonicum* | Not effective | 38.3% | 61% | 31% | 86.7 |
| B- The ability of at 100 mg/kg as a single oral dose **303** to kill juvenile worms in an *in vivo* study (5 animals were treated with a single dose by oral gavage with 100 mg/kg) | | | | | |
| Day post infection | | 20 | 25 | 28 | 32 |
| Reduction in worm burden | | NS | 63.8% | 48.9% | 54.1% |
| C- Combination treatment of PZQ and OXA derivatives against PZQ-resistant strain (5 animals were treated with a single dose by oral gavage with 100 mg/kg of each) | | | | | |
| Combination treatment | | PZQ + **610** | | PZQ + **303** | |
| Reduction in worm burden | | 52.9 % | | 90.8% | |
